# Evaluation of Eosinophilic Cationic Protein as a Marker of Alveolar and Cystic Echinococcosis

**DOI:** 10.3390/pathogens11020261

**Published:** 2022-02-18

**Authors:** Julian Frederic Hotz, Klaus Kaczirek, Stefan Stremitzer, Fredrik Waneck, Herbert Auer, Thomas Perkmann, Manuel Kussmann, Philipp Karl Bauer, Rui-Yang Chen, Richard Kriz, Heinz Burgmann, Michael Ramharter, Heimo Lagler

**Affiliations:** 1 Department of Medicine I, Division of Infectious Diseases and Tropical Medicine, Medical University of Vienna, A-1090 Vienna, Austria; julian.hotz@hotmail.de (J.F.H.); manuel.kussmann@meduniwien.ac.at (M.K.); philipp.bauer@meduniwien.ac.at (P.K.B.); rui-yang.chen@meduniwien.ac.at (R.-Y.C.); richard.kriz@meduniwien.ac.at (R.K.); heinz.burgmann@meduniwien.ac.at (H.B.); 2 Department of Internal Medicine III, Division of Infectious Diseases, University Hospital of Ulm, D-89081 Ulm, Germany; 3 Department of General Surgery, Medical University Vienna, A-1090 Vienna, Austria; klaus.kaczirek@meduniwien.ac.at (K.K.); stefan.stremitzer@meduniwien.ac.at (S.S.); 4 Department of Biomedical Imaging and Image-Guided Therapy, Division of Cardiovascular and Interventional Radiology, Medical University of Vienna, A-1090 Vienna, Austria; frederik.waneck@meduniwien.ac.at; 5 Department of Medical Parasitology, Institute of Specific Prophylaxis and Tropical Medicine, Center of Pathophysiology, Infectiology and Immunology, Medical University of Vienna, A-1090 Vienna, Austria; herbert.auer@meduniwien.ac.at; 6 Department of Laboratory Medicine, Medical University of Vienna, A-1090 Vienna, Austria; thomas.perkmann@meduniwien.ac.at; 7 Department of Tropical Medicine, Bernhard-Nocht-Institute for Tropical Medicine & I, Department of Medicine, University Medical Center Hamburg-Eppendorf, D-20359 Hamburg, Germany; ramharter@bnitm.de

**Keywords:** echinococcosis, eosinophilic cationic protein, routine marker

## Abstract

Echinococcosis is a neglected zoonotic disease and a worldwide public health problem caused by infection with the larval stages of taeniid cestodes of the genus *Echinococcus*. In vitro studies have demonstrated a protoscolecidal effect of eosinophilic cationic protein (ECP), a granule protein of eosinophilic granulocytes, against *E. granulosus*. Therefore, the main objective of this study was to evaluate ECP as a biomarker in the treatment of alveolar echinococcosis (AE) and cystic echinococcosis (CE). Data were collected retrospectively from the Vienna Echinococcosis Cohort over 7 years until December 2020. Altogether, 32 patients (16 AE and 16 CE) were included. In the selected patients, serum ECP values were compared before and after the beginning of an operative and/or benzimidazole (BMZ) therapy. Mean ECP serum levels before intervention were significantly (*p* < 0.05) elevated at 34.0 ± 22.9 μg/L in AE patients and at 38.6 ± 19.9 μg/L in CE patients compared to the control group. After the intervention, mean ECP levels decreased significantly (*p* < 0.05) to 20.4 ± 14.6 μg/L in AE patients and to 22.4 ± 8.3 μg/L in CE patients. Furthermore, ECP showed a significant (*p* < 0.05) correlation of k = 0.56 with PET–CTI. Based on the significant decrease after operative and/or BMZ treatment and the correlation with clinical markers such as PET–CTI, it is recommended to investigate ECP more intensively as a marker of AE and CE in prospective studies with larger cohorts.

## 1. Introduction

Echinococcosis is a zoonosis caused by infection with a tapeworm of the genus *Echinococcus*. An infection with *E. granulosus* leads to cystic echinococcosis (CE); an infection with *E. multilocularis* leads to alveolar echinococcosis (AE). CE is characterized by mostly unilocular cysts and AE by multiple smaller lesions [1]. AE is predominantly found in the temperate to cold latitudes of the Northern Hemisphere [1,2]. The main endemic areas are Southern Germany, Western Austria, Eastern France, Northern Switzerland, Russia, China, and the Japanese island Hokkaido. Due to the increased urbanization of foxes, and probably also due to the increased use of immunosuppressants, the numbers of AE have risen steadily in recent years [3,4]. By comparison, CE is not restricted to the Northern Hemisphere. The main endemic areas are in South America, Eastern Europe, the Middle East, East Africa, Central Asia, China, and Russia [5].

The prognosis of patients with AE has improved considerably in recent decades, partly due to better imaging, surgical, and drug procedures [5]. Therapy should be considered according to the PNM classification (P = parasitic mass in the liver, N = involvement of neighboring organs, and M = metastasis), based on cross-sectional images [6]. Benzimidazoles (BMZ) such as albendazole, or alternatively mebendazole, are available for drug therapy and should be given to every AE patient as a first line therapy. Curative surgical treatment followed by BMZ therapy should be evaluated for every patient. AE patients must be observed and treated for several years. For serodiagnosis of AE, a sensitive ELISA such as *Echinococcus* IgG or immunoblotting tests are usually the first-line investigation, followed by more specific tests for *E. multilocularis* such as recEm18-ELISA or Em2+-ELISA, which can lead to differentiation between *E. multilocularis* and *E. granulosus* by using specific parasitic antigens [7,8]. For the serodiagnosis of CE, the sensitivity of serological makers may differ depending on the localization of the cysts [5,9]. Except these specific serological markers, there are only very few markers for routine controls, such as total immunoglobulin E (IgE) or eosinophil granulocyte counts. Routine markers can serve as the first indication of an infection with echinococcosis. The role of total IgE has hitherto been controversial. It is frequently used as a routine marker and is therefore already highly evaluated. At the time of initial presentation, eosinophilia in the blood is more likely to be seen in CE and is rarely found in AE. All in all, the established markers’ total IgE and eosinophil granulocyte counts are limited due to their low sensitivity in CE, but even more limited in AE [10]. The eosinophil cationic protein (ECP) could represent a new promising routine marker measured in serum.

ECP, together with major basic protein (MBP), eosinophil peroxidase, and eosinophil-derived neurotoxin (EDN), is a granule of eosinophil granulocytes [11]. As are other members of the ribonuclease A family, the gene that encodes ECP is on chromosome 14 [12]. The molecular weight variates between 16 and 24 kDa and it is extremely basic, with a pI of pH 10.8 [13]. ECP exhibits potent immunomodulatory, antibacterial, antiparasitic, and antiviral properties, and here is cytotoxic to a wide bandwidth of pathogens [13,14]. Originally developed to assess progression in patients with severe type I allergy, it is associated with many diseases, e.g., asthma, cancer, or infectious diseases such as malaria [15,16,17,18]. It should be mentioned that there are phases in the etiopathology when the parasite cannot completely control the immunological response of the host. At that time, an increase in the eosinophil count and thus a local degranulation can take place. This could be the reason why ECP and EDN become present inside hydatid cysts in analog concentrations compared to reported concentrations in allergic disorders [19,20]. Previously, recombinant human ECP (rhECP) has showed toxic effects against protoscoleces (PSC) and the germinal layer of *E. granulosus*. Dose-dependent concentrations of rhECP led to progressive instability and finally disintegration of the tegument. ECP proved to be more toxic against *E. granulosus* than EDN. This resulted in the consideration that it is not the ribonuclease activity, but the cationic structure of the ECP that leads to its toxicity [20]. In a clinical analysis, a correlation was found between the symptoms and the ECP levels in patients with CE [21].

This retrospective pilot study evaluated ECP as a potential clinical routine marker of AE and CE, and considered ECP for further prospective studies with larger cohorts due to its in vitro protoscolecidal effects against *E. granulosus*.

## 2. Materials and Methods

### 2.1. Inclusion and Exclusion Criteria

The data from the Vienna Echinococcosis Cohort were used for the analysis. This cohort includes patients with AE and CE treated at the Medical University of Vienna at the Clinical Department for Infections and Tropical Medicine. This cohort was established to create a database in Austria for future studies. Between 1 January 2014 and 31 December 2020, the Vienna Echinococcosis Cohort added 28 new patients with confirmed AE; of those, n = 16 AE patients were included in the data analysis. Furthermore, *n* = 16 CE patients were examined from the cohort. Included were patients who had undergone intervention in the form of a drug or surgical therapy and pre- and post-interventional examinations at Vienna General Hospital, Austria, during the previously mentioned time period. Exclusion criteria were patients with confirmed allergies (AE: *n* = 0, CE: *n* = 1) and missing pre- and/or post-interventional ECP values (AE: *n* = 12, CE: *n* = 11). In addition, a total of *n* = 19 patients initially suspected of having AE or CE were included in the analysis. These patients were discussed during the interdisciplinary echinococcosis board of the Medical University of Vienna consisting of specialists in infectious diseases, visceral surgery, and radiology. Here, echinococcosis was excluded based on anamnestic, serological, radiological, and/or histological criteria. The data evaluation was approved and accepted by the Ethics Committee of the Medical University of Vienna in June 2020 under the EK number 2031/2012. All patients of the Vienna Echinococcosis Cohort were asked for venous blood sampling at the beginning of therapy and at subsequent follow-up appointments. If consent was given, a 10 mL serum tube was taken every appointment and ECP was routinely measured for all AE and CE patients. The one excluded CE patient with confirmed allergies had permanent ECP values outside the range of >200 μg/L and was excluded to maintain the quantitative data quality. Participation in this study had no direct benefit for the patient, but no risk was expected since it was a retrospective data evaluation.

### 2.2. Demographic Data

The data analysis included 16 patients with confirmed AE (group A), 16 patients with CE (group B), and 19 patients in the control group (group C). The gender distribution in group A was balanced with 8 (50.0%) male and 8 (50.0%) female patients, whereas in group B there were more male (m/w: 1.5) and in group C more female patients (m/w: 0.46). The mean age at the time of the first blood sampling was 53.9 ± 18.4 years (20–80) in group A. As expected, group B was significantly (*p* < 0.05) younger with 40.3 ± 13.6 years (18–62). The control group was on average 54.6 ± 17.2 years (22–82) old.

In group A, 13 patients (81.25%) received a disease-specific operation followed by BMZ drug therapy. Three patients (18.75%) received palliative drug therapy with BMZ. All patients had a liver manifestation and, in line with the literature, 4 patients (25.0%) already had parasitic lesions in other organs such as the lungs, pleura, and perisplenic or adrenal glands [2]. The preinterventional ECP values were taken at a median of 2.5 months (Q1: 0.5, Q3: 3.8) before the intervention. The postinterventional measurement was the first clinical appointment that included blood samples after the intervention, hereupon the values were taken at a median of 4.2 months (Q1: 1.2; Q3: 6.0) in the operative and at a median of 7.6 months (4.9–9.3) after the intervention in the BMZ therapy scheme.

A total of 10 patients (62.5%) from group B underwent a disease-specific operation followed by BMZ drug therapy. Six patients (37.5%) received palliative drug therapy. Excepting three patients, all patients had liver cysts. Patients without hepatic manifestations had extrahepatic cysts in the uterus, adnexa, right thigh, and lung (Table 1). The ECP values of CE patients were taken at a median of 0.7 months (Q1: 0.2, Q3: 2.8) before the intervention. In the operative therapy scheme, the blood samples were collected at a median of 2.0 months (Q1: 0.7, Q3: 3.6) and in the BMZ therapy scheme 4.1 months (Q1: 1.9, Q3: 6.5) after the intervention.

In group C, all patients were initially suspected to have AE or CE. In the further interdisciplinary investigations, 9 patients were diagnosed with simple liver cysts, 3 patients with splenic cysts, 2 patients with carcinoma and 1 patient with hemangioma. For 4 patients, the diagnosis remained unclear, but it was certainly not echinococcosis.

### 2.3. Interventions and Imaging

During the entire observation period, the patients received one or more interventions in the form of a disease-specific operative therapy and/or drug therapy using benzimidazoles. Throughout this time, the therapy was oriented based on the WHO suggestions [5]. For patients included in the operative therapy scheme, R0-resections were used.

Out of group A, 15 patients received a staging PET–CTI shortly before the intervention. The tracer uptake was categorized as either none, weak, or strong. AE Patient 11 instead underwent an MRI before the intervention because adrenocortical carcinoma was assumed present; for that reason, only the lesion size is included in the data analysis. Every CE patient obtained a cross-sectional image (CTI, MRI, or PET) before the intervention. The mean lesion sizes were 35.5 ± 21.2 cm^2^ in AE patients and 53.1 ± 37.7 cm^2^ in CE patients. On the basis of the cross-sectional images or ultrasound, AE patients were classified using the PNM classification and CE patients using the Gharbi et al. expanded WHO-IWGE CE cyst classification [5,22,23,24]. If a CE patient had two or more cysts at different stages of activity, the more dominant cyst was used for the data analysis. Not all CE patients received their PET–CTI as a cross-sectional image; for that reason, there was no data analysis with tracer uptake in CE patients.

### 2.4. ECP Measurement

ECP was measured in an ISO 9001:2015 certified and ISO 15189:2012 accredited laboratory (Department of Laboratory Medicine, Medical University of Vienna, Austria) using the CE-IVD ImmunoCAP™ECP test on a fully automated Phadia 250 analyzer (both Thermo Fisher Scientific, Immunodiagnostics, Vienna, Austria). The eosinophilic cationic protein was released in vitro during the coagulation process from prestimulated and activated eosinophils. For the best possible standardization of ECP release in vitro, serum tubes with gel separator were allowed to clot for a minimum of 60 and a maximum of 120 min before centrifugation. After centrifugation, the serum was transferred to a new tube and stored temporarily at 2–8 degrees Celsius until measurement. The ECP test had a calibration of from 2–200 µg/L, the analytical sensitivity was 0.5 µg/L, and the coefficients of variation (intra- and inter-assay) were typically from 4–6%. According to the 95th percentile of apparently healthy individuals, values <13.3 µg/L are considered normal.

### 2.5. Statistical Analysis

The data were analyzed using SPSS version 27.0. Normal distribution was checked by using a Kolmogorov–Smirnov test. If a normal distribution was present, parametric tests were used for group comparisons. If there was no normal distribution, non-parametric tests were used. For dependent variables, this was the Wilcoxon test. For the evaluation, the confidence interval was set at 95% and the significance level at 0.05. The null hypothesis assumed that there was no difference between the ECP values before and after an intervention.

## 3. Results

### 3.1. Eosinophilic Cationic Protein in Patients with Alveolar Echinococcosis

Before the intervention, mean ECP serum levels were elevated at 34.0 ± 22.9 μg/L, and were thus significantly (*p* < 0.05) higher than in patients out of the control group (mean 14.4 ± 7.7 μg/L). The lowest preinterventional ECP value was 8.1 μg/L and the highest was 79.8 μg/L. Overall, 13 patients (81.3%) showed preinterventional elevated ECP levels above the norm (<13.3 μg/L). Patients over 65 years (*n* = 5) had preinterventional ECP levels of 39.3 ± 25.1 μg/L. The patient collective between 55 and 65 years (*n* = 5) showed an average ECP level of 27.7 ± 23.7 μg/L and the collective under 55 years (*n* = 6) showed an average ECP level of 34.8 ± 23.4 μg/L. There was no significant (*p*_1_ = 0.47, *p*_2_ = 0.25, *p*_3_ = 0.86) difference in the ECP values between the age groups. The patients in the operative therapy scheme (*n* = 13) had a mean ECP level of 37.8 ± 23.2 μg/L preinterventionally. The average ECP level of the three patients with benzimidazole only was 17.3 ± 14.3 μg/L.

**Figure 1:**
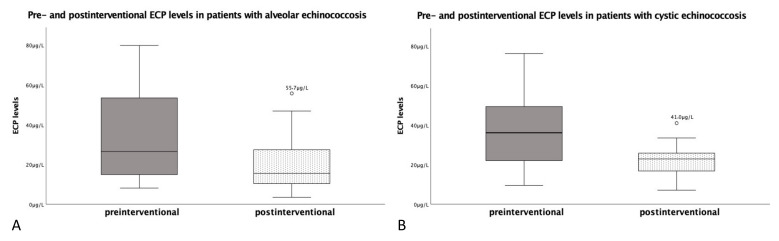
Pre- and post-interventional ECP levels in patients with alveolar or cystic echinococcosis. (**A**) shows the ECP values of AE patients before and after a disease-specific intervention, while (**B**) illustrates those of the CE patients.

Postinterventionally, the mean ECP levels showed a significant (*p* < 0.05) decrease to 20.4 ± 14.6 μg/L (Figure 1A). The lowest postinterventional ECP level was 3.5 μg/L and the highest was 55.7 μg/L. A total of 11 patients (68.8%) still had ECP levels above 13.3 μg/L (Table 1). Additionally, patient 13 showed a strong increase in ECP value after an intervention. It should be mentioned that in the next PET–CTI, several small suspicious and new foci in the liver were detected. Patients with an operative treatment (*n* = 13) had an average ECP level of 23.5 ± 14.5 μg/L postoperatively. The patients in the drug therapy schema (*n* = 3) had an average ECP level of 7.2 ± 5.4 μg/L at a median of 7.6 months after beginning benzimidazole therapy.

Furthermore, ECP showed a significant (*p* < 0.05) correlation of k = 0.56 with tracer uptake in PET–CTI. There was no significant (*p*_1_ = 0.39, *p*_2_ = 0.12) correlation between the PNM classification and the parasitic lesion size (Table 2).

### 3.2. Eosinophilic Cationic Protein in Patients with Cystic Echinococcosis

Preinterventionally, mean ECP serum levels were elevated at 38.6 ± 19.9 μg/L and hence were significantly (*p* < 0.05) higher than in the control group (14.4 ± 7.7 μg/L). The lowest ECP value was 9.5 μg/L and the highest was 76.2 μg/L. A total of 15 patients (93.8%) showed elevated ECP levels above the norm (<13.3 μg/L). There was no significant (*p* = 0.49) difference between the ECP values of CE and AE patients.

Postinterventionally, the mean ECP levels showed a significant (*p* < 0.05) decrease to 22.4 ± 8.3 μg/L (Figure 1B). The lowest postinterventional ECP level was 7.1 μg/L and the highest was 41.0 μg/L. It should be pointed out that the ECP value of patient 28 dropped further in upcoming follow-ups to 5.9 μg/L and subsequently showed an ECP increase of up to 23.0 μg/L. Radiologically, a hepatic recurrence was confirmed.

For patients with CE, there was no correlation between the ECP value and the lesion size (*p* = 0.52) or WHO-IWGE CE cyst activity (*p* = 0.49).

## 4. Discussion

Patients with echinococcosis, especially with AE, are usually followed and treated for many years. Therefore, it is essential to find markers for initial diagnosis and follow-up strategies. In this retrospective data analysis and pilot study, the aim was to evaluate ECP as a potential marker for routine controls of AE and CE. Previous routine markers such as total IgE or eosinophil granulocyte counts give first indications of a parasitic infection. However, their relevance is controversial; therefore, it is important to find further routine markers that could be helpful when thinking about a parasitic disease and to subsequently use specific serological markers. Furthermore, they could help to detect a recurrence in blood controls outside of special echinococcosis clinics. 

ECP has been associated with CE in previous studies [20,21]. Cicioğlu et al. showed that mean ECP levels in patients with CE were significantly higher than levels in the control group, whereas no differences in ECP levels were shown for age or gender. In accordance with this, there was no significant difference in ECP levels between the age groups in our data analysis. Furthermore, ECP already showed a response to the clinical condition of the patient, as CE patients with symptoms had significantly higher ECP levels than asymptomatic CE patients [21]. In our data analysis, there was no sign of a correlation (*p* = 0.52) between the radiological parameter lesion size and ECP values in patients with CE. 

So far, there are no publications that link ECP with AE. Due to the increasing incidences in Central Europe and the few available routine markers, additional markers should be detected and integrated into the routine. In our data analysis, mean ECP serum levels before intervention were elevated at 34.0 μg/L ± 22.9 in AE patients and at 38.6 μg/L ± 19.9 in CE patients. After the intervention, mean ECP levels in AE patients decreased significantly (*p* < 0.05) to 20.4 μg/L ± 14.6 and in CE patients to 22.4 ± 8.3 μg/L. The ECP values of both diseases appeared significantly (*p* < 0.05) increased in comparison with the control group. The AE patient population used in this data analysis was not optimal due to their distribution into 13 operative and 3 drug-only therapy schemes. Usually, AE therapy is more often a palliative BMZ therapy than a surgical therapy [25]. Larger studies could therefore look at the behavior of ECP in surgical treatment and drug therapy separately. Here, it would be essential to see how quickly ECP levels decrease after R0 resection, as in our analysis, 76.9% operative AE patients and all operative CE patients had elevated ECP levels; a rather slow decrease became apparent. Therefore, a possible explanation could be that ECP reflects the patient’s immune response rather than the presence of parasite antigen detected with specific ELISAs or direct pathogen detection. Supporting this explanation, a significant (*p* < 0.05) correlation of k = 0.56 between the ECP and the PET–CTI was found in our data analysis. Thus, a rapid decrease into negative values is rather not to be expected after surgical and/or BMZ therapy. Additionally, there were large time differences in the collection of postoperative serologies in our data analysis. 

It is important to note that CE cysts can be subdivided according to the WHO-IWGE classification [5]. Ramos et al. found out that the hydatid cyst walls of inactive CE5 cysts with signs of calcifications have a higher ECP content than the hydatid cyst walls of active CE1 cysts [20]. Whether this plays a role only histopathologically in terms of a strong localized immune response or is also detectable in peripheral blood should be evaluated in larger cohorts, since no significant (*p =* 0.49) correlation could be detected in our limited cohort. However, it is suggested that dead parasites are more potent triggers of a strong immune response than living parasites [26]. In line with this, patient 24 showed constantly slightly elevated ECP values above the cut-off until the occurrence of a rupture of the cyst, followed by an increase of up to 50.2 μg/L.

Furthermore, future studies could detect what impact palliative BMZ therapy has on ECP. BMZ therapy is a long-term palliative treatment where the effects on specific serological ELISAs or immune response markers are not expected to be immediate after the start of therapy [27]. However, it should be examined how ECP compares to specific serological ELISAs such as Em^2+^, rec-Em18, or *Echinococcus* IgG, since as mentioned before both ECP and PET–CTI are more likely to represent the immune response than specific serological parameters, which are more likely to reflect parasitic metabolism [28].

In conclusion, based on the significant decrease of ECP serum levels after operative and/or BMZ treatment in AE and CE patients and its correlation with tracer uptake in PET–CTI in AE patients, it is recommended to investigate ECP more intensively as a marker of AE and CE in prospective studies with larger cohorts.

## Figures and Tables

**Table 1:** Demographic data and ECP levels.

**Patients**	**Gender**	Age *	**Disease**	**PNM** **Classification**	**Therapy Scheme**	**Organ Manifestation**	**ECP** **Preinterventional**	**ECP** **Postinterventional**	**Lesion** Size **	**Traceruptake in PET-CTI**
**Group A**										
patient 1	female	80	AE	P3N0M1	BMZ	liver, perisplenic	9.9 μg/L	4.9 μg/L	57.6 cm^2^	strong
patient 2	female	27	AE	P3N0M0	operative + BMZ	liver	28.9 μg/L	10.4 μg/L	70.3 cm^2^	none
patient 3	female	59	AE	P2N0M0	operative + BMZ	liver	15.2 μg/L	55.7 μg/L	23.4 cm^2^	none
patient 4	female	20	AE	P2N0M0	operative + BMZ	liver	38.5 μg/L	14.1 μg/L	18.4 cm^2^	weak
patient 5	male	68	AE	P2N0M0	BMZ	liver	33.7 μg/L	13.4 μg/L	26.0 cm^2^	weak
patient 6	male	60	AE	P1N0M0	operative + BMZ	liver	8.1 μg/L	10.4 μg/L	4.0 cm^2^	none
patient 7	male	57	AE	P2N0M0	BMZ	liver	8.3 μg/L	3.5 μg/L	17.1 cm^2^	none
patient 8	female	21	AE	P2N1M0	operative + BMZ	liver, pleura	14.6 μg/L	16.6 μg/L	12.3 cm^2^	none
patient 9	female	36	AE	P2N0M0	operative + BMZ	liver	24.1 μg/L	16.9 μg/L	34.3 cm^2^	weak
patient 10	male	72	AE	P3N0M0	operative + BMZ	liver	57.1 μg/L	24.6 μg/L	28.2 cm^2^	weak
patient 11	male	71	AE	P4N0M1	operative + BMZ	liver, lung, adrenal	72.0 μg/L	28.1 μg/L	45.5 cm^2^	-
patient 12	male	66	AE	P3N0M0	operative + BMZ	liver	24.0 μg/L	31.8 μg/L	23.2 cm^2^	weak
patient 13	male	55	AE	P3N0M0	operative + BMZ	liver	56.4 μg/L	14.4 μg/L	48.0 cm^2^	weak
patient 14	female	63	AE	P3N0M0	operative + BMZ	liver	50.5 μg/L	8.7 μg/L	70.5 cm^2^	strong
patient 15	male	54	AE	P3N0M0	operative + BMZ	liver	79.8 μg/L	46.9 μg/L	24.5 cm^2^	strong
patient 16	female	53	AE	P4N0M1	operative + BMZ	liver, lung	22.6 μg/L	22.7 μg/L	64.0 cm^2^	weak
**Patients**	**Gender**	Age *	**Disease**	CE Cyst Classification ***	**Therapy Scheme**	**Organ Manifestation**	**ECP** **Preinterventional**	**ECP** **Postinterventional**	**Lesion Size**	
**Group B**										
patient 17	male	48	CE	CE2	operative + BMZ	liver, lung	58.7 μg/L	24.9 μg/L	70.5 cm^2^	
patient 18	female	34	CE	CE2	operative + BMZ	liver	76.2 μg/L	41.0 μg/L	36.3 cm^2^	
patient 19	male	45	CE	CE5, CE1	BMZ	liver	44.8 μg/L	14.9 μg/L	60.9 cm^2^	
patient 20	female	32	CE	CE1	operative + BMZ	liver	28.3 μg/L	24.7 μg/L	127.8 cm^2^	
patient 21	male	40	CE	CE3a	operative + BMZ	right thigh	48.5 μg/L	26.7 μg/L	40.1 cm^2^	
patient 22	male	51	CE	CE2	BMZ	liver	33.0 μg/L	7.1 μg/L	25.4 cm^2^	
patient 23	male	27	CE	CE1	operative + BMZ	liver	17.1 μg/L	31.0 μg/L	63.2 cm^2^	
patient 24	male	30	CE	CE3a	operative + BMZ	lung	50.2 μg/L	33.5 μg/L	5.9 cm^2^	
patient 25	female	62	CE	CE1	operative + BMZ	liver, lung	36.2 μg/L	18.8 μg/L	108.2 cm^2^	
patient 26	male	20	CE	CE3a	operative + BMZ	liver	41.7 μg/L	16.1 μg/L	16.4 cm^2^	
patient 27	male	54	CE	CE3a	BMZ	liver, lung, spleen	75.5 μg/L	22.7 μg/L	116.5 cm^2^	
patient 28	female	42	CE	CE2, CE3b	operative + BMZ	uterus, adnexa	23.9 μg/L	17.5 μg/L	71.5 cm^2^	
patient 29	male	58	CE	CE3a	BMZ	liver	36.0 μg/L	23.5 μg/L	34.0 cm^2^	
patient 30	female	18	CE	CE4, CE5	BMZ	liver, kidney	9.5 μg/L	13.3 μg/L	13.7 cm^2^	
patient 31	female	30	CE	CE3b, CE1	operative + BMZ	liver, lung	20.2 μg/L	19.9 μg/L	25.8 cm^2^	
patient 32	female	54	CE	CE1	BMZ	liver	17.2 μg/L	23.1 μg/L	33.2 cm^2^	

* Age in years at the time of the first blood sampling; ** Two largest parasitic lesions in two-dimensional space; *** WHO-IWGE CE cyst classification [22].

**Table 2:** Correlations between clinical parameters and ECP in AE patients.

			PNM Classification	Tracer Uptake in PET–CTI	Lesion Size
Spearman Rho	**Alveolar Echinococcosis**				
	ECP *	k **	0.23	0.56	0.40
		p	0.39	*p* < 0.05	0.12
		*n*	16	15	16

* preinterventional ECP values; ** correlation coefficient.

## Data Availability

The data presented in this study are available on request from the corresponding author. The data are not publicly available due to ethical concerns and data privacy reasons.
